# Antibiotic Resistance of Bacteria

**DOI:** 10.1155/2015/501658

**Published:** 2015-08-04

**Authors:** Madhab K. Chattopadhyay, Ranadhir Chakraborty, Hans-Peter Grossart, Gundlapally S. Reddy, Medicharla V. Jagannadham

**Affiliations:** ^1^Centre for Cellular and Molecular Biology, (CSIR), Hyderabad 500 007, India; ^2^Molecular Microbiology Laboratory, Department of Biotechnology, University of North Bengal, Siliguri, West Bengal 734 430, India; ^3^Experimental Limnology, Leibniz Institute of Freshwater Ecology and Inland Fisheries (IGB), Alte Fischerhuette 2, 16775 Stechlin, Germany; ^4^Institute of Biochemistry and Biology, Potsdam University, Am Neuen Palais 10, 14469 Potsdam, Germany

Antibiotic resistance of bacteria and other microorganisms is one of the most serious and grievous challenges of the twenty-first century. The life-saving drugs, which held a great deal of promises during the 1940s to eradicate all the infectious life-threatening diseases in the world, have ceased to work, because of the increasing emergence of microbial strains invulnerable to them. Many of the previously efficacious antibiotics are no longer usable because of widespread occurrence of multiresistant microbial strains. Lately, discovery of new antibiotics is failing to keep pace with the emergence of (multi)resistance of pathogenic and also environmental bacterial strains. Consequently, the prospect of chemotherapy looks bleak. The trepidation that we might be pushed back to a situation analogous to the preantibiotic era, when no chemotherapeutic agent was available to contain and combat deadly bacterial infections, does not appear to be an overblown imagination.

Based on this backdrop, this special issue appears to be an aptly undertaken and well-timed endeavour to address this global problem. The articles contributed by investigators from various research laboratories with different scientific backgrounds have not only portrayed the width of the problem but also displayed some silver lining in the management of the looming crisis. Rapid detection of the profile of resistance is essential for timely application of the right antibiotic to a patient. H. Frickmann et al. summarize the efficacy and limitations of various molecular and mass spectrometric methods for the detection of resistance. The omnipresent nature of the resistant organisms is revealed in a number of articles. F. B. Atique and Md. M. R. Khalil report on the occurrence of antibiotic resistance among bacteria (predominantly skin commensal coagulase-negative staphylococci) isolated from allogenic bone samples for grafting, collected from different hospitals of Bangladesh. Food materials are believed to serve as a vehicle for transmission of resistance. This issue is addressed by F. S. Dehkordi et al. who report on the genotype and resistance-profile of* Helicobacter pylori* isolated from vegetables and salad samples, picked up from groceries and supermarket in a province of Iran. The high similarity in the genotype pattern of the isolates obtained from vegetables and humans indicates transmission. A. B. Flórez et al. reveal tetracycline and erythromycin-resistant bacteria and genes conferring resistance to these antibiotics in 10 Spanish and 10 Italian samples of commercial cheese. P. Krupa et al. report on the population structure (based on* spa* typing) of oxacillin-resistant* Staphylococcus aureus *isolated from nasal swabs of pigs, collected from two slaughter houses of Poland. Some meat samples bought from the shops were also included into their studies. D. De Vito et al. characterize multidrug-resistant clinical isolates of* Salmonella typhimurium* for resistance genes in an area of southern Italy by pulsotyping and phage typing. C. Zhang et al. report on the resistant phenotype and genotype of* Streptococcus suis* serotype 2, isolated from 62 clinically healthy sows and 34 diseased pigs reared in different farms of China. Antibiotic resistance in the nosocomial isolates is a matter of serious concern. F. Lombardi et al. look into the molecular epidemiology of carbapenemase-producing strains of* Klebsiella pneumoniae* isolated from the surgery unit at a cardiovascular centre of Italy. D. Ojdana et al. demonstrate the ability of an* E. coli* strain obtained from a hospital of Poland to produce carbapenemase enzymes and also the presence of genes responsible for the production of carbapenemases and other *β*-lactamases. Extended-spectrum-*β*-lactamase (ESBL) is a bacterial enzyme having the ability to hydrolyse even the third-generation cephalosporins and aztreonam. Besides* Klebsiella pneumoniae *some strains of* Escherichia coli* are also known to produce this enzyme. This is indicated by M. S. Rezai et al. who performed genotyping of ESBL-producing strains of* E. coli*, obtained from a paediatric hospital of north Iran. The authors also show the association of ESBL-positive* E. coli* strains with resistance to various other antimicrobials. Occurrence of ESBL-producing Enterobacteriaceae in iceberg lettuce obtained from the retail market of Rochester (US) is described by N. Bhutani et al. A wide spectrum of diseases is caused by the virulent strains of ESBL-positive isolates of* E. coli*. Regional difference in the prevalence of virulence genes in 432 phenotypically ESBL-positive patient-isolates of* E. coli *(obtained from the Baltic Sea region) is shown by J. Lillo et al. Keeping in mind the tremendous challenge posed by drug-resistant tuberculosis, a number of relevant articles are included in this collection. The susceptibility profile of* M. tuberculosis* isolates to various antitubercular antibiotics varies significantly depending on the test system as revealed by Z. Mei et al. They have also shown that changes in bacterial susceptibility are further caused by mixed infection with particular genotypes of* M. tuberculosis* strains. Resistance-profile of 100 strains of* M. tuberculosis*, isolated from patients in northeast Iran, is reported by A. T. Sani et al. Occurrence of nontuberculosis* Mycobacterium,* in 25 out of 125 patients (20%) surveyed, underscores the need of proper diagnosis before the onset of chemotherapy.

Discovery of new drugs and strategies to circumvent antibiotic resistance is the need of the hour to contain the problem. N. Jafari et al. report on the isolation of an antibiotic-producing strain of a soil Actinomycetes belonging to the genus* Pseudonocardia*. The antibacterial compound produced by it is effective against* Staphylococcus aureus*. They have also purified and partially characterized this compound. R. D. Wojtyczka et al. demonstrate high antibacterial activity of two new quinoline derivatives of a structure of 3-thioacyl 1-methyl 4-arylaminoquinolinium salts against some nosocomial strains of staphylococci in both planktonic and biofilm form. In view of the widespread nature of the problem caused by inefficacy of the antibiotics produced by fermentation and chemical synthesis, it is necessary to tap alternative sources (e.g., plant kingdom) for novel antibiotics. P. Del Serrone et al. demonstrate antibacterial activity of Neem seed oil (*Azadirachta indica* A. Juss.) against enteropathogenic strains of* E. coli* and indicate that some of the ciprofloxacin-resistant isolates lost their virulence following treatment with Neem seed oil. Antimicrobial peptides are considered potential candidates for the management of multidrug-resistant infections. M. Singh and K. Mukhopadhyay evaluate the antimicrobial potential of an anti-inflammatory neuropeptide whereas C. Chen et al. report on the efficacy of recombinant lysostaphin against methicillin-resistant* S. aureus* (MRSA) in a mouse model. Widespread use of carbapenems is associated with emergence of resistance. The polymyxin antibiotic colistin is not used at present because of its nephrotoxicity. H.-J. Tang et al., however, demonstrate the efficacy of a combination of colistin and imipenem against carbapenem-resistant* Klebsiella pneumoniae*. Bacteriophages could be suitable alternatives for antibiotics, which currently have lost efficacy because of the emergence of resistant strains. N. Shivshetty et al. demonstrate the potential of a bacteriophage isolated from sewage to protect diabetic mice against* Pseudomonas aeruginosa*-induced bacteremia. Reversal of bacterial resistance to antibiotics is essential to restore the efficacy of the existing antimicrobials. C. Santiago et al. claim to achieve an increase in susceptibility of a MRSA strain to ampicillin when it was combined with a plant extract. A number of computerized models have been developed during the recent past to assist the physicians with the necessary information to enable prescription of the right antibiotic in the right moment. M. Rodriguez-Maresca et al. report on the efficacy of a new electronic device based on laboratory data on the most probable susceptibility profile of pathogens responsible for infections and also on local epidemiology.

